# Bariatric surgery: pre-operative comorbidities, postoperative results, and complications: a single-center retrospective cohort analysis

**DOI:** 10.1590/0102-67202025000034e1903

**Published:** 2025-10-27

**Authors:** Raphael Sidney BANDEIRA, Kaio Waltrick VIEIRA, Beatriz Schuelter TREVISOL, Fabiana SCHUELTER-TREVISOL, Daisson José TREVISOL

**Affiliations:** 1 Universidade do Sul de Santa Catarina, Graduate Program in Health Sciences – Tubarão (SC), Brazil.; 2 Universidade do Sul de Santa Catarina, Medical School – Tubarão (SC), Brazil.

**Keywords:** Bariatric Surgery, Metabolic Syndrome, Obesity, Weight Loss, Postoperative Complications, Cirurgia Bariátrica, Síndrome Metabólica, Obesidade, Redução de Peso, Complicações Pós-Operatórias

## Abstract

**Background:**

Bariatric surgery is the most effective treatment for weight loss and also promotes remission of preoperative metabolic comorbidities.

**Aims:**

The aim of this study was to analyze preoperative comorbidities, evaluate postoperative outcomes, and assess complications 6 months after bariatric surgery in a hospital in the state of Santa Catarina, Brazil.

**Methods:**

A retrospective cohort study was conducted with patients who underwent bariatric surgery between 2021 and 2022 and were followed up for a period of 6 months after the procedure.

**Results:**

There was a predominance of female patients (81.6%), with a mean age of 38.7 years. The preoperative prevalence of hypertension, Type 2 diabetes, dyslipidemia, and hepatic steatosis was 36.7, 22.4, 22.4, and 32.7%, respectively. The postoperative remission rates for these conditions were 55, 64, 70, and 69%, respectively. Except for diabetes, no significant differences were found between the Roux-en-Y gastric bypass (RYGB) and sleeve gastrectomy (SG) groups. There was a significant reduction in weight (p<0.01) and body mass index (BMI) (p<0.01), with no statistical differences between the RYGB and SG groups. Postoperative complications occurred in 73.5% of patients, including anemia, vitamin deficiencies, cholelithiasis, dumping syndrome, anastomotic ulcer, chronic diarrhea, and anastomotic stricture.

**Conclusions:**

The study described the preoperative comorbidity profile, postoperative outcomes, and complications with findings consistent with existing literature, except for underreporting dyslipidemia and hepatic steatosis. No statistical difference was observed between the surgical techniques performed.

## ARTICLE HIGHLIGHTS

Bariatric surgery led to significant remission of metabolic comorbidities such as hypertension (55%), Type 2 diabetes (64%), and dyslipidemia (64%) within 6 months after the procedure.

There was significant weight loss among operated patients, with an average reduction of 32.5 kg and a body mass index (BMI) decrease of 12.1 kg/m2, regardless of the surgical technique used (Roux-en-Y gastric bypass [RYGB] or sleeve gastrectomy [SG]).

More than 70% of the patients experienced some postoperative complications, most frequently nutritional deficiencies (iron, vitamin D, and B12) and dumping syndrome, with similar rates across surgical techniques.

The study reinforces the short-term effectiveness of bariatric surgery but highlights the need for strict postoperative follow-up due to the high prevalence of adverse effects and the potential underdiagnosis of preexisting comorbidities.

## Summary

**CENTRAL MESSAGE:**

Bariatric surgery, whether through Roux-en-Y gastric bypass (RYGB) or vertical sleeve gastrectomy (SG), is associated with significant short-term weight loss and remission of metabolic comorbidities in patients with morbid obesity. This retrospective cohort study conducted in southern Brazil reinforces the effectiveness of both surgical techniques and highlights the importance of structured postoperative monitoring due to the high frequency of nutritional deficiencies and other complications, even in the early months following surgery.

**PERSPECTIVES:**

Clinical Implication: Both Roux-en-Y gastric bypass (RYGB) and sleeve gastrectomy (SG) resulted in substantial weight loss and remission of comorbidities such as hypertension, Type 2 diabetes, dyslipidemia, and hepatic steatosis within 6 months, with no statistically significant differences between techniques, apart from the selective allocation of diabetic patients to the RYGB group. Public Health Relevance: In the context of the rising obesity burden in Brazil and worldwide, bariatric surgery remains a critical intervention not only for weight reduction but also for improving metabolic health, with direct implications for reducing long-term morbidity and healthcare costs. Future Research: Longer follow-up studies with prospective designs and comprehensive data collection are needed to assess long-term outcomes and late complications and to better differentiate the efficacy of surgical techniques beyond selection biases.

## VISUAL ABSTRACT

**Figure:**
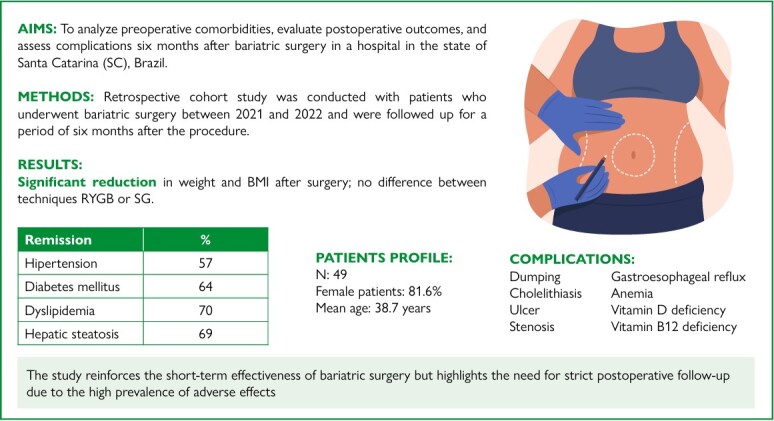

## RESUMO

**Racional:**

A cirurgia bariátrica é o tratamento com melhor perda de peso, além de remissão de comorbidades metabólicas pré-operatórias.

**Objetivos:**

Analisar as comorbidades pré-operatórias, avaliar o resultado pós-operatório e complicações associadas após 6 meses da cirurgia bariátrica em um hospital do estado de Santa Catarina, Brasil.

**Métodos:**

Estudo de coorte retrospectiva com os pacientes submetidos á cirurgia bariátrica no período 2021 a 2022 e que tiveram um acompanhamento por um período de seis meses após o procedimento.

**Resultados:**

Houve uma predominância do sexo feminino (81,6%), a idade média foi de 38,7 anos e a prevalência pré-operatória de hipertensão arterial, diabetes tipo 2, dislipidemia, esteatose hepática foram, respectivamente, de 36,7; 22,4; 22,4 e 32,7%. As taxas de remissão pós-operatória destas doenças foram 55; 64; 70 e 69%. Exceto a diabetes, não houve diferença entre os grupos submetidos a *bypass* em Y de Roux (BYR) e gastrectomia vertical (GV). Houve queda significativa do peso (p<0,01) e do índice de massa corpórea (IMC) (p<0,01), mas sem diferenças entre os grupos BYR e GV. Complicações pós-operatórias ocorreram em 73,5% dos indivíduos, incluindo anemia, deficiência de vitaminas, colelitíase, dumping, úlcera de boca anastomótica, diarreia crônica e estenose de anastomose.

**Conclusões:**

O estudo traçou um perfil de comorbidades pré-operatórias, resultados pós-operatórios e complicações com achados compatíveis com a literatura existente, exceto pela subnotificação de dislipidemia e esteatose. Não houve diferença estatística quanto ao procedimento realizado.

## INTRODUCTION

 Obesity is a chronic disease with a rising incidence that affects a large proportion of the population and is associated with morbidity and mortality. Its incidence has been increasing in most countries, reaching approximately 878 million adults in 2022, an increase of 684 million compared to 1990. This prevalence shows significant disparities across different regions, reaching rates as high as 70% in American Samoa^24^. In Brazil, obesity is projected to affect 29.8% of the population by 2030^9^. According to the World Health Organization, obesity is considered the second leading preventable cause of death after smoking, associated with increased risks of hypertension, diabetes, and cancer^3,16,26^ . 

 In this epidemic context, bariatric surgery has been increasingly indicated. Long-term studies have identified bariatric surgery as the most effective treatment for weight loss compared to non-surgical approaches^1,13^. This therapeutic option is also associated with high rates of remission of metabolic comorbidities such as hypertension, Type 2 diabetes, dyslipidemia, and sleep apnea^14^. The remission of Type 2 diabetes stands out as the most significant, with rates reaching 60–80%^28^. Currently, the most commonly performed techniques are sleeve gastrectomy (SG) and Roux-en-Y gastric bypass (RYGB), accounting for 90% of procedures^2^. However, this therapeutic approach is not free of complications. Complications may be classified as early, occurring in the first weeks, or later, appearing months or years after the procedure. Among early complications, the most prevalent are anastomotic leakage, intraoperative bleeding, surgical site infection, and venous thromboembolism. Among late complications, nutritional deficiency and dumping syndrome are noteworthy, with the latter being more associated with RYGB^6^. 

 There are few studies on the follow-up and incidence of complications related to bariatric surgery in the state of Santa Catarina. The aim of this study was to evaluate preoperative comorbidities, postoperative outcomes, and associated complications 6 months after bariatric surgery in a hospital in the state of Santa Catarina, Brazil. 

## METHODS

 A retrospective cohort study was conducted with patients who underwent bariatric surgery for morbid obesity at a hospital in southern Santa Catarina. Data were obtained through medical record analysis. 

### Study population and sampling

 Patients who underwent surgery for morbid obesity at Hospital Nossa Senhora da Conceição (HNSC) between 2021 and 2022 and had medical follow-up within 6 months after the procedure were evaluated. 

### Inclusion and exclusion criteria and ethical aspects

 A total of 63 patients underwent bariatric surgery and attended follow-up appointments with the medical team during the first 6 months after the procedure was included in this study. However, 14 participants were excluded due to incomplete or insufficient medical record data that prevented the study objectives from being addressed. 

 The study was previously submitted to the Research Ethics Committee (CEP) of the institution and was approved under protocol no. 5.918.319, issued by the Plataforma Brasil in March 2023. Furthermore, the study was conducted in accordance with the guidelines and regulatory standards for research involving human subjects, as outlined in Resolution No. 466/2012 of the Brazilian National Health Council. 

### Variables and data collection

 The variables collected included sex, age, surgical technique used, weight before and after bariatric surgery, body mass index (BMI) pre- and postoperatively, comorbidities (dyslipidemia, hypertension, diabetes mellitus, hepatic steatosis, and sleep apnea) before and after the procedure, and postoperative complications. 

 Data were obtained through the analysis of patient medical records, provided by the responsible medical team and healthcare professionals at HNSC. The collected data were entered into a Microsoft Excel^®^ spreadsheet containing all the study variables. 

### Data processing and analysis methods

 The data were organized and analyzed using Jamovi software (version 2.3.28). Quantitative variables were described using measures of central tendency and data dispersion. To assess differences between groups, Student’s t-test was applied for quantitative variables. Qualitative variables were described using absolute and relative frequencies. Differences in proportions were tested using Fisher’s exact test. The level of statistical significance adopted was 5% (p<0.05). 

## RESULTS

A total of 49 participants were included in the study, and the preoperative characteristics are presented in Table 1.

**Table 1:** Clinical and epidemiological characteristics of patients undergoing bariatric surgery at a hospital in southern Brazil, 2021/2022, before surgery.

Characteristics		RYGB	SG	p-value
Sex, n (%)				
	Male	7 (19.4)	2 (15.4)	1.0*
	Female	29 (80.6)	11 (84.6)	
Age (mean±SD)				
	Years	39.1±12.8	37.5±8.8	0.671†
Hypertension, n (%)				
	Yes	14 (38.9)	4 (30.8)	0.743*
	No	22 (61.1)	9 (69.2)	
Diabetes mellitus, n (%)				
	Yes	11 (30.6)	0 (0)	0.024*
	No	25 (69.4)	13 (100.0)	
Dyslipidemia, n (%)				
	Yes	10 (27.8)	1 (7.7)	0.246*
	No	26 (72.2)	12 (92.3)	
Hepatic steatosis, n (%)				
	Yes	13 (36.1)	3 (23.1)	0.502‡
	No	23 (63.9)	10 (76.9)	
Sleep apnea, n (%)				
	Yes	1 (2.8)	1 (7.7)	0.464*
	No	35 (97.2)	12 (92.3)	
Hypothyroidism, n (%)				
	Yes	3 (8.3)	3 (23.1)	0.321*
	No	33 (91.7)	10 (76.9)	
Anxiety, n (%)				
	Yes	4 (11.1)	1 (7.7)	1.0‡
	No	32 (88.9)	12 (92.3)	
Depression, n (%)				
	Yes	3 (8.3)	2 (15.4)	0.598‡
	No	33 (91.7)	11 (84.6)	

RYGB: Roux-en-Y gastric bypass; SG: sleeve gastrectomy; SD: standard deviation.

* Student’s t-test for quantitative variables

† Mean±SD or number (%)

‡ Fisher’s exact test for qualitative variables and calculated using

 There was a predominance of the female sex (81.6%), with 80.6% in the bypass group (RYGB) and 84.6% in the SG group. No statistically significant difference was found between the groups (p=1.0, p>0.05). Regarding age, the mean was 38.7 years (range 21–67 years); the mean age in the RYGB group was 39.1 years, similar to the SG group (37.5 years), with no significant difference (p=0.671, p>0.05). 

 Concerning the presence of comorbidities, systemic arterial hypertension was present in 36.7% of the patients, with a similar distribution between groups (38.9% in the RYGB group and 30.8% in the SG group). Diabetes mellitus was present in 22.4%, all of whom belonged to the RYGB group, representing 30.6% of that group. Dyslipidemia accounted for 22.4% of the total sample, occurring more frequently in the RYGB group (27.8 versus 7.7%), but without a statistically significant difference (p=0.246, p>0.05). Hepatic steatosis was observed in 32.7% of the patients, with an equivalent distribution between the RYGB and SG groups (36.1 versus 23.1%, respectively). Obstructive sleep apnea was recorded in two participants (one in each group). 

 Hypothyroidism was found in 12.2% of patients (8.3% in the RYGB group and 23.1% in the SG group) but without a significant difference (p=0.321, p>0.05). Anxiety disorder was observed in 10.2% of the patients (11.1% in the RYGB group and 7.7% in the SG group), with no significant difference. Major depressive disorder was recorded in 10.2% (8.3% in the RYGB group and 15.4% in the SG group), also without a statistically significant difference (p=0.598, p>0.05). 

 Other reported diseases included polycystic ovary syndrome (one case in the RYGB group), Crohn’s disease (one case in the SG group), gastroesophageal reflux disease (three cases in the RYGB group), cardiac arrhythmia (one case in the RYGB group), and asthma (one case in the RYGB group). 

### Evaluation of weight loss

 Comparing the weight in kilograms (kg), there was a significant reduction in both weight (p<0.01) and BMI (p<0.01) when comparing preoperative and postoperative data for all patients. In the subgroup analysis, significant reductions were also observed in both the RYGB and SG groups. However, when comparing the percentage of weight loss in RYGB and SG subgroups, a greater percentage of weight loss was noted in the RYGB group (30.6% versus 27.6%), although this difference was not statistically significant (p=0.095, p>0.05) (Table 2). 

**Table 2:** Evaluation of postoperative weight loss according to surgical technique.

Measured parameters		RYGB	SG	All patients
Weight (kg)				
	Preoperative	109±14.4	106±8.59	108±13.1
	Postoperative	75.1±11.2	76.6±11.7	75.5±11.2
	Mean difference	34.5	28.2	32.5
	p-value*	<0.01	<0.01	<0.01
	Effect size	3.62	3.04	3.46
BMI (kg/m^2^)†				
	Preoperative	40.9±4.2	38.5±2.61	40.2±4
	Postoperative	28.1±3.8	28.0±4.2	28.1±3.9
	Mean difference	13.3	10.3	12.1
	p-value*	<0.01	<0.01	<0.01
	Effect size	3.49	2.73	3.33
% Weight loss†		30.8±7.1	27.6±8.6	30.0±7.5

RYGB: Roux-en-Y gastric bypass; SG: sleeve gastrectomy; BMI: body mass index.

* Student’s t-test for quantitative variables

† Mean value±standard deviation (SD) for each group.

### Evaluation of remission of metabolic comorbidities

 In the evaluation of remission of preexisting comorbidities, there was a reduction in the prevalence of hypertension from 36.7 to 16.3% when comparing the preoperative and postoperative periods. This represents a significant remission of 55% (p=0.002, p<0.05), with remission rates of 57% in the RYGB group and 50% in the SG group. Regarding diabetes, there was a reduction in prevalence from 22.4 to 8.1%, representing a 64% remission (all diabetic patients were in the RYGB group). In the evaluation of dyslipidemia, an overall remission rate of 64% was also observed ,which was statistically significant (p=0.008, p<0.05), with 70% remission in the RYGB group. Among patients with hepatic steatosis, there was also significant remission (69%, p<0.01), with similar distribution between subgroups (69% in the RYGB group and 67% in the SG group). For the two patients with obstructive sleep apnea, no remission was reported (Table 3). 

**Table 3:** Pre- and postoperative comorbidities according to group and surgical technique.

Assessed comorbidities		RYGB	SG	All patients (%)
Hypertension				
	Preoperative	14	4	18 (36.7)
	Postoperative	6	2	8 (16.3)
	% Remission*	57	50	55.0
	p-value†			0.002
Diabetes mellitus				
	Preoperative	11	0	11 (22.4)
	Postoperative	4	0	4 (8.1)
	% Remission*	64	-	64.0
	p-value†			0.008
Dyslipidemia				
	Preoperative	10	1	11 (22.4)
	Postoperative	3	1	4 (8.1)
	% Remission*	70	0	64.0
	p-value†			0.008
Hepatic steatosis				
	Preoperative	13	3	16 (32.7)
	Postoperative	4	1	5 (10.2)
	% Remission*	69	67	69.0
	p-value†			<0.001
Sleep apnea				
	Preoperative	1	1	2 (4.1)
	Postoperative	1	1	2 (4.1)
	% Remission*	0	0	0
	p-value†			-

RYGB: Roux-en-Y gastric bypass; SG: sleeve gastrectomy.

* Number (percentage)

† McNemar’s test

### Evaluation of postoperative complications

 Of the total participants, 73.5% experienced some type of complication, including anemia, vitamin deficiency, cholelithiasis, dumping syndrome, marginal ulcer, chronic diarrhea, and anastomotic stenosis. There was no difference between the groups (72.2% in the RYGB group versus 76.9% in the SG group, p>0.05). Table 4 shows the percentages of each type of complication. When analyzing each complication separately, no significant differences were observed between the groups. 

**Table 4:** Adverse events by group and surgical technique

Complications	RYGB* (%)	SG (%)	p-value†
Dumping	7 (19.4)	4 (30.8)	0.451
Cholelithiasis	7 (19.4)	0 (0)	0.167
Ulcer	2 (5.6)	1 (7.7)	1.0
Stenosis	1 (2.8)	1 (7.7)	0.464
Gastroesophageal reflux	1 (2.8)	1 (7.7)	0.464
Anemia	5 (13.9)	1 (7.7)	1.0
Vitamin D deficiency	8 (22.2)	3 (23.1)	1.0
Vitamin B12 deficiency	6 (16.7)	2 (15.4)	1.0

RYGB: Roux-en-Y gastric bypass; SG: sleeve gastrectomy.

* Number (percentage)

† Fisher’s exact test.

 Nutritional deficiencies were observed in 40.8% of the patients who underwent gastroplasty (46.2% in the SG group versus 38.9% in the RYGB group, p=0.648, p>0.05). The deficiencies identified were iron-deficiency anemia, vitamin D deficiency, and vitamin B12 deficiency. No significant difference was observed between the groups. 

 Another reported complication was related to a patient with Crohn’s disease who presented with epiploic appendagitis and sigmoiditis, in addition to chronic diarrhea (SG group). 

## DISCUSSION

 Analyzing the epidemiological characteristics of participants undergoing bariatric surgery, we observed similarities regarding the profile described in most studies. In this study, there was a predominance of the female sex. It is known that women are more affected by obesity (15 of women versus 11% of men), but men are more likely to have comorbidities and present comorbidities with proportionally lower BMI than women^19^. 

 The average age of patients in this study was 38.7 years. Individuals over 50 years of age tend to have a higher prevalence of comorbidities and a greater tendency for increased hospital stay after bariatric surgery. Furthermore, younger patients undergoing bariatric surgery tend to have a higher BMI than those over 60 years of age^4^. 

 The prevalence of hypertension is increased in obese patients compared to normal-weight individuals (40 versus 15%)^18^, similar to what was found in this study (36.7%) in obese patients. Regarding the prevalence of diabetes, it was 22.4%, similar to a British study reporting 19.1%^31^. The prevalence of dyslipidemia in obese patients is 60–70%^10^, a value much higher than the 22.4% found in the present study, indicating underdiagnosis of this condition. The prevalence of hepatic steatosis was 32.7%, much lower than a similar Brazilian study^23^ which reported 76.3%, possibly also indicating underdiagnosis. Other diseases related to obesity include polycystic ovary syndrome^29^, obstructive sleep apnea^30^, and cancer^5^; patients with the first two conditions were identified in this study. The rate of obese patients preoperatively diagnosed with obstructive sleep apnea by polysomnography is 70%, with 40% being severe cases^30^. This rate is much higher than that found in the present study, possibly indicating underdiagnosis. Regarding group division, we observed similarity between the two groups (RYGB and SG), except for the presence of diabetes. This selection bias is due to studies^21,25^ recommending RYGB as the gold standard for diabetic patients, hence the indication of this procedure for this target population. 

 Regarding weight loss, the expected weight loss reported in the literature is up to 40% in the first 6 months, stabilizing over the following 18 months and reaching an average of 25% of the initial weight^17^. The value found in this study (30%) is within the expected range. It is known that SG has good weight loss results and, despite other studies^ 21,30^, this research showed no statistical difference compared to RYGB. A possible explanation is selection bias since all diabetic patients were in the RYGB group. Future studies may demonstrate whether excluding diabetic patients results in SG showing similar outcomes to RYGB. 

 A recent literature review on hypertension remission and bariatric surgery^22^ showed remission rates around 50%. In our study, we found a 55% remission of hypertension. Conflicting studies do not clarify whether one technique is superior to the other (RYGB versus SG). In this study, similar values were found between both techniques. Diabetes remission rates vary from 23% to 60% associated with weight loss of 20–30% after metabolic surgery^15^. In this study, a remission rate of 64% was found. This occurs because of important alterations in glucose metabolism, including increased hepatic insulin sensitivity, decreased hepatic glucose production and hepatic triglycerides, increased insulin sensitivity in adipose tissue, increased intestinal peptide hormones such as glucagon-like peptide-1 (GLP-1) and peptide YY (PYY), and increased bile acid production^28^. Most of these changes occur with both surgical techniques (RYGB or SG), but some changes, such as intestinal morphology alterations, are associated only with RYGB^28^. Based on a meta-analysis^30^, most surgeons recommend RYGB in diabetic patients due to higher remission rates, influencing the allocation of all patients with this pathology to the RYGB group. The remission rate of hepatic steatosis in our study was 69%, while dyslipidemia remission reached 70%, values consistent with literature reporting up to 83%^8^. Although weight loss decreases the severity of obstructive sleep apnea, only 37% of patients with this condition achieve total remission^17^ after bariatric surgery, consistent with our findings, where no remission of this disease was observed. 

 Dumping syndrome affects a wide range of gastrectomized patients (1–75%), with a higher frequency in patients undergoing RYGB^7^. It results from the sudden presence of gastric contents in the proximal small intestine and is associated with the release of bradykinin, serotonin, and enteroglucagon^7^. In our study, dumping was found in 19.4% of the RYGB group and 30.8% of the SG group, without a statistical difference. The risk of cholelithiasis is increased after bariatric surgery due to obesity and rapid weight loss, potentially reaching rates up to 50%^20^. It is more commonly associated with RYGB^26^ and showed an incidence of 19.4% in this subgroup in our study. Anastomotic ulcer was recorded in 4.6% of patients undergoing RYGB^27^, with higher risk in smokers and users of nonsteroidal anti-inflammatory drugs. In this study, the incidence was 5.6% in this subgroup, but ulcers were present in both groups (RYGB and SG) without a statistical difference. Anastomotic stenosis is a complication found in 6%–20% of patients undergoing RYGB^12^ but was found in 2.8% in this subgroup in our study. Post-SG stenosis is considered a rare complication involving 0.26%–4% of procedures^11^, but in our series, it was found in 7.7%. The incidence of new gastroesophageal reflux is reported in up to 20% of patients undergoing SG, while RYGB is considered a protective barrier against this complication^27^. In our study, 4% of the patients experienced this complication, with one case reported in each group (SG and RYGB). Iron-deficiency anemia, vitamin D deficiency, and vitamin B12 deficiency are alterations found in post-bariatric surgery with frequencies up to 55%, 100%, and 20%, respectively^27^. In this study, anemia was found in 12.2%, vitamin D deficiency in 22.4%, and vitamin B12 deficiency in 16.3% of the patients, with no statistical difference between the RYGB and SG groups. 

 The main limitations of this study were the short followup period (6 months) and the use of retrospective data limited to what was recorded in medical charts, which may lead to underreporting. Despite these limitations, this study demonstrated that patients undergoing bariatric surgery present excellent postoperative results in weight loss and remission of comorbidities. Additionally, this type of procedure requires appropriate postoperative follow-up due to frequent postoperative complications. 

## CONCLUSIONS

 The study recorded preoperative comorbidities, postoperative outcomes, and complications consistent with the literature, except for underreporting dyslipidemia and hepatic steatosis among patients undergoing bariatric surgery, at a hospital in southern Santa Catarina. No statistical difference was found regarding the surgical procedure performed (RYGB or SG), except that all diabetic patients were allocated to the RYGB group. 

## Data Availability

The information regarding the investigation, methodology, and data analysis of the article is archived under the responsibility of the authors.
